# MicroRNA-182-5p Attenuates Asthmatic Airway Inflammation by Targeting NOX4

**DOI:** 10.3389/fimmu.2022.853848

**Published:** 2022-05-31

**Authors:** Zhiguang Wang, Yilan Song, Jingzhi Jiang, Yihua Piao, Li Li, Qiaoyun Bai, Chang Xu, Hanye Liu, Liangchang Li, Hongmei Piao, Guanghai Yan

**Affiliations:** ^1^ Jilin Key Laboratory for Immune and Targeting Research on Common Allergic Diseases, Yanbian University, Yanji, China; ^2^ Department of Respiratory Medicine, Affiliated Hospital of Yanbian University, Yanji, China; ^3^ Department of Anatomy, Histology and Embryology, Yanbian University Medical College, Yanji, China; ^4^ Department of Intensive Care Unit, Affiliated Hospital of Yanbian University, Yanji, China

**Keywords:** miR-182-5p, NOX4, airway inflammation, asthma, Ovalbumin (OVA)

## Abstract

Bronchial asthma is characterized by chronic airway inflammation, airway hyperresponsiveness, and airway remodeling. MicroRNA (miRNA) has recently been implicated in the pathogenesis of asthma. However, the mechanisms of different miRNAs in asthma are complicated, and the mechanism of miRNA-182-5p in asthma is still unclear. Here, we aim to explore the mechanism of miRNA182-5p in asthma-related airway inflammation. Ovalbumin (OVA)-induced asthma model was established. MiRNA Microarray Analysis was performed to analyze the differentially expressed miRNAs in the asthma model. We found that the expression of miRNA-182-5p was significantly decreased in OVA-induced asthma. *In vitro*, IL-13 stimulation of BEAS-2B cells resulted in a significant up-regulation of NOX4 (nicotinamide adenine dinucleotide phosphate oxidase 4), accompanied by mitochondrial damage-induced apoptosis, NLRP3 (NOD-like receptor family pyrin domain-containing 3)/IL-1β activation, and reduced miRNA-182-5p. In contrast, overexpression of miRNA-182-5p significantly inhibited epithelial cell apoptosis and NLRP3/IL-1β activation. In addition, we found that miRNA-182-5p could bind to the 3’ untranscripted region of *NOX4* mRNA and inhibit epithelial cell inflammation by reducing oxidative stress and mitochondrial damage. *In vivo*, miRNA-182-5p agomir treatment significantly reduced the percentage of eosinophils in bronchoalveolar lavage fluid, and down-regulated Th2 inflammatory factors, including IL-4, IL-5, and OVA induced IL-13. Meanwhile, miRNA-182-5p agomir reduced the peribronchial inflammatory cell infiltration, goblet cell proliferation and collagen deposition. In summary, targeting miRNA-182-5p may provide a new strategy for the treatment of asthma.

## Introduction

Bronchial asthma is a chronic airway inflammation. Currently, nearly 45.7 million people suffer from asthma, and the number of patients is increasing (1). Th1/Th2 imbalance is the main pathogenesis of asthma, and about 50% of adult asthma patients have eosinophilia and Th2 airway inflammation. In the presence of co-activators (such as epithelial-derived thymic stromal lymphopoietin), Th2 will produce inflammatory cytokines, such as IL-5, IL-4, and IL-13, after allergic sensitization and subsequent stimulation of dendritic cells (2). IL-13 promotes goblet cell proliferation and mucus production and accelerates airway smooth muscle proliferation and collagen deposition. All these can cause airway remodeling, leading to a significant increase in airway responsiveness (3, 4). Moreover, IL-13 induces the apoptosis of bronchial epithelial cells, promotes the expression of a-SMA (alpha-smooth muscle actin) and type III collagen, and finally induces airway fibrosis (5). Inflammasomes are a family of multiprotein signaling complexes and nucleotide-binding oligomerization domain (NOD)-like receptors (NLR), of which NLRP3 (NOD-like receptor family pyrin domain-containing 3) inflammasomes are particularly important in the pathogenesis of asthma. Deficiency of NLRP3 could reduce Th2 activation and decrease secretion of Th2 cytokines IL-5, IL-13 as well as chemokines (6). Targeting the NLRP3 inflammasome is therefore also the key to the treatment of asthma.

Oxidative stress refers to the increase of reactive oxygen species (ROS) under a variety of factors, resulting in oxidative damage to proteins and nucleic acids and inducing disease. Oxidative stress can promote the pathogenesis of asthma. Excessive ROS expression can damage cellular structures such as carbohydrates, nucleic acids, and proteins and change their function, leading to pathological changes in respiratory epithelial cells, increased vascular and tracheal permeability, excessive mucus secretion, excessive smooth muscle cell contraction, and airway hyperreactivity through a series of regulatory mechanisms (7, 8). Meanwhile, asthmatic Th2-type inflammatory factors can similarly regulate ROS generation. IL-4 induces IgE release from B cells, binds to the receptor Fcele on mast cells, and regulates ROS generation. IL-4, IL-5, and IL-13 can regulate H_2_O_2_-mediated dual oxidase activity involved in ROS regulation. It can be seen that Th2-type inflammatory factors interact with oxidative stress and aggravate the asthmatic response (9). NOX4 (nicotinamide adenine dinucleotide phosphate oxidase 4), as one of the nicotinamide adenine dinucleotide phosphate oxidase (NOX) family, catalyzes the reduction of molecular oxygen to H_2_O_2_ and is responsible for the generation of ROS, which enables NOX4 to perform special functions in different cells, including involvement in proliferation, differentiation, apoptosis, and angiogenesis (10). NOX4 has been implicated in a variety of respiratory diseases, including acute respiratory distress syndrome (11), chronic obstructive pulmonary disease (COPD) (12), lung cancer (13), and asthma (14). NOX4 is not only involved in ciliary motility dysfunction in asthmatic epithelial cells, but also aggravates oxidative stress load in airway smooth muscle and aggravates airway hyperresponsiveness (14, 15). ROS have been identified as an important NLRP3 inflammasome activator in various diseases (such as hepatic ischemia/reperfusion injury) (16). Given that NOX4 can regulate the expression of ROS, NOX4 may also be involved in regulating NLRP3 activation in asthma. However, this has not yet been reported. In addition, mitochondria are another important organelle for ROS production. Mitochondrial ROS (mtROS) is a double-edged sword that regulates cellular function (17), and high levels of mtROS resulting from mitochondrial damage inevitably can lead to the oxidation of DNA, proteins, and lipids (18). In asthma, mtROS can act as signal transducer to trigger the expression or release of inflammatory cytokines and aggravate the inflammatory response, while mitoTEMPO, a mitochondria-specific antioxidant, has been shown to improve airway inflammation in asthmatic mice, indicating that mitochondria are also involved in the pathogenesis of asthma (19). Studies have shown that abnormalities in mitochondrial dynamics (fission/fusion) lead to mitochondrial dysfunction, which is closely related to the development and progression of lung diseases, including asthma (20, 21). Mitochondrial dysfunction secondary to defects in mitochondrial dynamics is known to lead to increased ROS generation, which further activates the mitochondria-dependent apoptotic pathway (22). *In vitro* and *in vivo* models of COPD have shown that cigarette smoke extract could lead to excessive apoptosis and dynamin-related protein 1 (Drp1) expression, while silencing Drp1 could reverse cigarette smoke extract-induced apoptosis (23). Thus, both oxidative stress and abnormal mitochondrial function are involved in the development of airway diseases.

MicroRNA (miRNA) is a non-coding RNA with a length of approximately 19-25 nucleotides, which is mainly directly involved in post-transcriptional regulation (24). Recent studies have shown that miRNAs have great application prospects as biomarkers for the diagnosis and efficacy evaluation of asthma (25, 26). MiRNA-182-5p is reported to be involved in many diseases. For example, overexpression of miRNA182-5p in a colon cancer model inhibited colon cancer tumorigenesis, angiogenesis, and lymphangiogenesis by directly down regulating vascular endothelial growth factor C (27). However, miRNA-182-5p expression was significantly increased in a mouse model of sepsis, which promoted intestinal inflammation by inhibiting the expression of surfactant protein D, and inhibition of miRNA-182-5P reduced apoptosis, promoted cell proliferation, and restored intestinal function (28). In the atherosclerosis model established by oxidized low-density lipoprotein, miRNA-182-5P ameliorated atherosclerosis by inhibiting oxidative stress and apoptosis by targeting toll-like receptor 4 (29). MiRNA-182-5p plays different roles in different diseases. However, the role and mechanism of miRNA-182-5p in asthma is poorly understood.

In this study, we used miRNA array to analyze differentially expressed miRNAs in the OVA-induced asthma model. We found that miRNA-182-5p was significantly down-regulated in asthma and was associated with airway inflammation. Through further *in vivo* and *in vitro* experiments, we investigated the role and mechanism of miRNA-182-5p in asthmatic airway inflammation. In previous studies, the miRNAs agomir, miRNA mimic, miRNA antagomir, and miRNA inhibitor have been transfected to *in vitro* and *in vivo* asthma models to achieve the effect of overexpressing or silencing miRNAs (30, 31). Thus, we used miRNA-182-5p mimic and miRNA-182-5p agomir to overexpress miRNA-182-5p *in vivo* and *in vitro*. Our finding may provide new therapeutic targets for the treatment of asthma in the future.

## Materials and Methods

### Mice

Female BALB/c mice (about 6-8 weeks old) with an average weight of (20 ± 2) g were from the Laboratory Animal Management Center of Yanbian University (Certificate No.: SYXK (Ji) 2020-0010). The humidity in the feeding environment was maintained at 50 ± 10% and the temperature was maintained at 20 ± 2°C. The animal experiment procedures were approved by the Ethics Committee of Yanbian University School of Medicine.

### Establishment of Asthma Model

Ovalbumin (OVA) induced asthma model was established in female BALB/c mice. Briefly, mice were sensitized with 200 μL of sensitization solution (OVA 10 μg + Al(OH)_3_ 1mg) by intraperitoneal injection on days 1, 7, and 14 (**Supplementary Figure 1**). From day 21, mice were challenged with 3% OVA nebulization for 30 min three times a week until day 56. For control, mice were intraperitoneally injected with 200 μL saline on days 1, 7, and 14, and were challenged with the same amount of normal saline from day 21 to day 56.

### MiRNA Microarray Analysis

Three mice with OVA induced asthma and three control mice were subjected to miRNA microarray analysis. They were euthanized on day 57 and lung tissues were collected. The total RNA was isolated from lung tissues and purified with TRIzol reagent (Invitrogen, Carlsbad, Canada) and an RNeasy Mini Kit (Qiagen, Hilden, Germany). RNA was detected with an UV–vis Spectrophotometer (Thermo, NanoDrop 2000, USA) at 260 nm. MicroRNA profiling was performed using the Multispecies miRNA 4.0 Array (Affymetrix GeneChip^®^, USA). Each group had three biological replicates. The RNA used in microarrays for microRNA was 130 ng. The microarrays were performed by Cnkingbio Biotechnology Corporation (Beijing, China). Data were analyzed with Robust Multichip Analysis algorithm using Affymetrix default analysis settings. Differentially expressed miRNAs between two groups were screened with p<0.05 and fold change >1.5. The microarray data has been uploaded into a public database.

### Animal Treatment and Grouping

Mice with OVA induced asthma were randomly divided into three groups (n=10/group), including OVA group, OVA + miRNA agomir NC (negative control) group, and OVA + miRNA-182-5p agomir group. Agomir NC and miRNA-182-5p agomir (20 nmol/mouse, RiboBio, Guangdong, China, Product No. MiR40000211-4-5), which is a miRNA duplex containing Robbio modifications such as 5 ‘sterol and 2’ OMe, were administered *via* tail vein twice a week from the start of the OVA nebulization challenge (day 21 to day 56) (**Supplementary Figure 1**). For control, normal mice (n=10) were administered with normal saline.

### Sample Collection

On day 57, the mice were anesthetized and sacrificed by inhalation of diethyl ether. The bronchoalveolar lavage fluid (BALF) was collected after intratracheal injection with 1mL pre-cooled normal saline. The recovery rate of BALF was required to reach 80% of the original normal saline. The collected BALF was centrifuged at 3000 r/min for 10 min at 4°C. The cell pellet was resuspended for subsequent flow cytometry to determine the number of eosinophils. The supernatant was used for subsequent cytokine detection. At the end of BALF collection, the thoracic cavity of mice was rapidly opened, the lung tissue of mice was extracted, and the left lung was fixed in 10% formaldehyde or cryopreserved.

### Histological Staining

Paraffin-embedded lung tissues were sectioned and stained with hematoxylin-eosin (HE), periodic acid-Schiff (PAS) and Masson to observe inflammatory cell infiltration, goblet cell proliferation and collagen deposition, respectively.

### ROS Detection

Cryopreserved tissues were cryo-sectioned and stained with Dihydroethidium (DHE) (Apexbio, USA), which is one of the most commonly used superoxide anion (O_2-_) fluorescence detection probes for cell membrane permeability and can be directly used for the detection of oxidative activity in living cells to observe tissue ROS expression. The DHE staining was performed as previously described (32). For intracellular ROS detection, BEAS-2B cells after treatment were incubated with DCFH-DA (10 μm/L, S0033S, Beyotime) for 20 min at 37°C and further analyzed by flow cytometry.

### Flow Cytometry Analysis of Eosinophils in BALF

After centrifugation, the cell pellet of collected BALF samples was re-suspended and labeled with leukocyte antibody CD45 + (#558702, BD, USA) and eosinophil antibody Siglec-F (#275639, Abcam, UK), respectively, at 4°C for 30 min. After washing, the percentage of eosinophils in BALF was determined by flow cytometry (Beckman Coulter, Inc., CA, USA).

### ELISA

The levels of IL-4, IL-5, IL-13 in BALF supernatant were determined using corresponding mouse ELISA kits (RampD Systems, Minneapolis, MN, USA) according to the manufacturer’s instructions.

### Cell Culture and Treatment

The human bronchial epithelial cell line BEAS-2B was purchased from the American Type Culture Collection (Rockville, MD, United States). They were cultured in DMEM containing 10% fetal bovine serum (Gibco BRL) and 1% penicillin/streptomycin (Gibco BRL) at 37°C with 5% CO2.

For IL-13 stimulation, human recombinant IL-13 (50 ng/ml, Stemcell, USA) was used to stimulate BEAS-2B cells for 48 h. MiRNA-182-5p mimic or NC (100 nM) (RiboBio, Guangdong, China) were transfected with Lipofectamine3000 (Invitrogen) for 24h followed by IL-13 stimulation. For the group pretreated with the inhibitor, cells were first incubated with the inhibitor Mdivi-1 (2.5 μM; Sigma) for 1 h before IL-13 stimulation. For siRNA transfection, cells were co-transfected with 1.5 µM of NOX4 siRNA (RiboBio, Guangdong, China) or NC siRNA (RiboBio, Guangdong, China) for 48 h. The silencing efficiency was verified by real-time PCR and Western blot.

### Real-Time PCR

Total RNAs and miRNAs from BEAS-2B cells and lung tissues of mice were extracted using Fastking Total RNA extraction kit (Tiangen, DP451, Beijing, China) or miRcute RNA extraction kit (Tiangen, DP501, Beijing). Then, 2 μg total RNAs and miRNAs were reverse transcribed with FastKing RT kit (Tiangen, KR118-02) and miR RT kit (Tiangen, KR211-02), respectively. Real-time PCR was performed with a three-step SYBR green RT-PCR kit (Tiangen, KR123) or miRcute RT-PCR kit (Tiangen, FP411-02) on the Azure cielo 6 system (Azure, Dublin, CA, USA). The primers of U6 and miRNA-182-5p and universal reverse primer were synthesized by and purchased from RiboBio (Guangdong, China). The NOX4 and GAPDH primers were purchased from Sangon Biotech (Shanghai, China). Primer sequences are as follows: NOX4, Forward CGTCTGGGCAGCTGAGTG, Reverse GAGCCAGATGAACAGGCAGA; miRNA-182-5p, UUUGGCAAUGGUAGAACUCACACCG; GAPDH, Forward TGCACCACCAACTGCTTAGC, Reverse GGCATGGACTGTGGTCATGAG; and, U6, Forward CTCGCTTCGGCAGCACA, Reverse AACGCTTCACGAATTTGCGT. The reaction condition for mRNA was: 95°C for 15min, followed by 40 cycles of 94°C for 10s, 55°C for 20s and 72°C for 20s. The amplification condition for miRNA was 95°C for 15min, followed by 45 cycles of 94°C for 20s and 55°C for 20s. Data were normalized to GAPDH or U6.

### Dual-Luciferase Reporter Assay

Based on predictions from the miRbase website (https://www.mirbase.org/), one potential binding site was found between *NOX4* 3′ untranscripted region (UTR) and miRNA-182-5p. Wild-type (WT) and mutant plasmids were constructed with pmiR-RB-ReportTM vector (RiboBio, Guangdong, China). HEK293 cells were co-transfected with plasmids (100 ng), and miRNA-182-5p mimic or NC (100 nM) using Lipofectamine3000 (Invitrogen, USA). Then, the luciferase activity assay was performed with the Dual-Luciferase Reporter Assay System (Promega, Madison, WI, USA) 48h after transfection using a GLOMAX96 spectrophotometer (Promega). The ratios of firefly (560 nm) and Renilla (480 nm) were measured separately for further analysis.

### The Extracellular Flux Analysis for Assessing Mitochondrial Stress

Mitochondrial function was analyzed using an extracellular flux analyzer (SeahorseXFp, Agilent, USA), and the results were expressed as ATP production, basal respiration, proton leak, spare respiration, maximum respiration, and non-mitochondrial oxygen consumption. Cells were treated in strict accordance with the manufacturer’s protocol. Briefly, cells were seeded in 8-well XF cell culture microplates (5000 cells/well) and stimulated with IL-13 and transfected with miRNA-182-5p mimic and miRNA-182-5p NC. Twenty-four hours before the assay, probe plates were hydrated overnight, and DMEM was adjusted to pH 7.4 with Seahorse basal medium (Agilent, USA). Cells were incubated in 200 μL DMEM for 1 h at 37°C in a CO2-free incubator. Then, 1.5 μM oligomycin, 2.0 μM FCCP, and 0.5 μM rotenone and antimycin a (Rot/AA) were added to SeahorseXF8 cell culture plates to obtain the relevant oxygen consumption rate (OCR). OCR values were analyzed after normalization by cell density using SeahorseWave software.

### Mitochondrial Morphology

BEAS-2B cells after stimulation were incubated with MitoTracker-Red (# M7512, Thermo Fisher) for 30 min at 37°C according to the manufacturer’s protocol. Mitochondrial morphology was photographed by the Cytation™ 5 (BioTek Instruments).

### Immunofluorescence Staining

Fluorescence staining of lung tissue and BEAS-2B cells was performed. Briefly, primary rabbit monoclonal antibody against NOX4 (ab133303, Abcam) used for incubation overnight at 4°C, followed by incubation with AlexaFluor488 goat anti-rabbit (A11001, Life Technologies, Waltham, MA, USA) for 2 h. BEAS-2B cells were stained with primary antibody against IL-1β (12703, CST) overnight at 4°C and then with AlexaFluor488 goat anti-rabbit (A11001, Life Technologies) for 2 h. Mitochondrial membrane potential (MMP) fluorescence staining was performed using tetramethylrhodamineethylester (TMRE) (113852, Abcam) at a final concentration of 50 nM at 37°C for 30 min. The mtROS fluorescence staining was performed using MitoSOX (M36008, mitoSOX mitochondrial superoxide Indicator, Thermo Fisher) at a final concentration of 5 μM at 37°C for 10 min. All data were collected by Cytation ™ 5 (BioTek Instruments).

### Apoptosis Assay

Apoptosis was measured using the AnnexinV-FITC Apoptosis Detection Kit (Beyotime, Shanghai, China) according to the manufacturer’s instructions. Cells were stained with Annexin V-FITC and propidium iodide (PI) for 30 min in the dark. Flow cytometer was used to detect the apoptosis rate of cells. All samples were detected with Cytoflex (Beckman Coulter, Inc., CA, USA) and analyzed using Cytoexpert 2.4 software.

### Western Blot

Western blot was used to analyze the expression of proteins. NOX4 (ab133303), Drp1 (ab184247), Bcl-2 (ab182858), cytochrome c (ab133504) and voltage-dependent anion channel 1 (VDAC1) (ab14734) antibodies were purchased from Abcam (Cambridge, MA, USA). Phosphorylated Drp1 (Ser616; p-Drp1s616) (#345), BAX (#5023), cleaved-caspase-9 (#20750), cleaved-caspase-3 (#9661), NLRP3 (#15101), ASC (Apoptosis-associated speck-like protein containing a C-terminal caspase recruitment domain) (#13833), Cleaved-caspase-1 (#89332), and IL-1β (#12703), β-actin (#4970) antibodies were purchased from CST (Danvers, MA, USA). β-actin was used as an internal control. Briefly, total protein was extracted, quantified by BCA protein quantification kit (Beyotime, Shanghai, China), separated by SDS-PAGE, and then transferred to nitrocellulose membrane. After blocking with skimmed milk, the membrane was incubated with the corresponding primary antibody overnight at 4°C. Incubation with the corresponding secondary antibody was performed for 2 h at room temperature. The gray density indicating relative protein levels was determined by Quantity One software (BioRad, Hercules, CA, USA).

In addition, the expression of Drp1 was examined in both the cytoplasm and mitochondria. Mitochondrial and cytosolic proteins were separated by a mitochondrial isolation kit (Beyotime, Shanghai, China) in strict accordance with the manufacturer’s instructions, and then analyzed by Western blot using VDAC1 and β-actin as the corresponding internal controls.

### Statistical Analysis

All results were analyzed using SPSS 22.0 software, and all graphs were plotted using Prism7.0. Data are presented as mean ± SD. Student *t*-test was used to compare between two groups, and one-way analysis of variance with Dunnett’s *post hoc* test was used to compare data among multiple groups. A *P* value < 0.05 was considered statistically significant.

## Results

### miRNA-182-5p Is Decreased in the OVA Asthma Model

**Figure 1:**
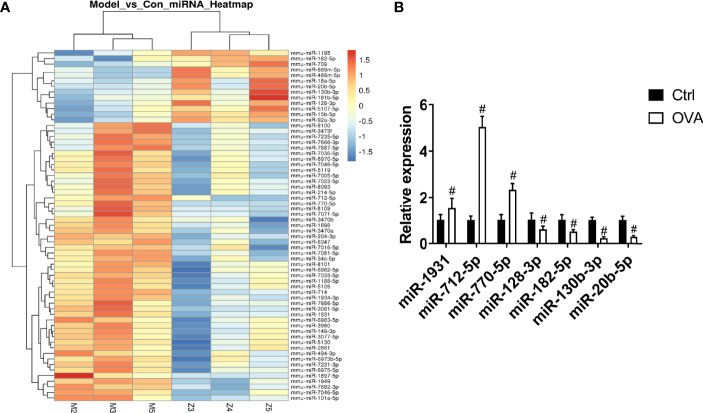
Comparison of miRNA differential expression in asthma models. **(A)** Hierarchical clustering of differential miRNAs. Red indicates up-regulated genes and green indicates down-regulated genes in asthma. **(B)** Real-time PCR was used to determine the expression changes of miR-1931, miR-712-5p, miR-770-5p, miR-128-3p, miR-182-5p, miR-130-3p, and miR-20b-5p in the Control and OVA groups. All data were shown as mean ± SD (n = 3). T-test was used for 2 group comparisons. #*p* < 0.05 versus control.

To identify the differentially expressed miRNAs in the pathogenesis of asthma, miRNA microarray analysis was performed. We found that 63 miRNAs were differentially expressed in the OVA group (*p* < 0.05), among which 50 miRNAs were upregulated, and 13 miRNAs were downregulated (**Figure 1A**). To further confirm the results of microarray analysis, we analyzed the levels of several miRNAs in lung tissues with real-time PCR. As shown in **Figure 1B**, compared with control group, the levels of miR-1931, miR-712-5p, and miR-770-5p were significantly increased in OVA group, while the levels of miR-128-3p, miR-182-5p, miR-130-3p, and miR-20b-5p were significantly decreased in OVA group. This was consistent with the microarray results. The miRNA-182-5p was one of the down-regulated miRNAs. Notably, miRNA-182-5p was reported to have an inhibitory effect on inflammation in other models (33, 34). However, its role and mechanism in asthma has been poorly studied. Thus, miRNA182-5p was further investigated in the following experiments.

### NOX4 Induces Airway Epithelial Inflammation by Mitochondrial Damage *via* ROS/Drp1

**Figure 2:**
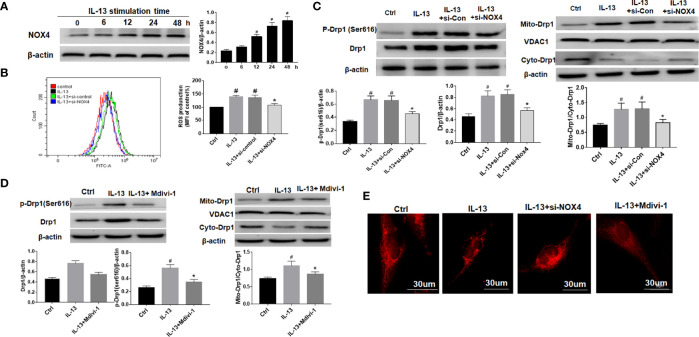
NOX4 induces airway epithelial inflammation by triggering mitochondrial damage *via* regulation of ROS/Drp1. **(A)** Western blot of NOX4 expression after IL-13 stimulation; **(B)** ROS expression was measured by flow cytometry, and NOX4 siRNA intervention was performed before IL-13 stimulation of BEAS-2B cells; **(C)** Western blot of Drp1 and p-Drp1 (Ser616) expression. **(D)** Western blot of Drp1 and p-Drp1 (Ser616) expression. Drp1 Inhibitor (Mdivi-1) intervention was conducted before IL-13 stimulation of BEAS-2B cells; **(E)** Mitochondrial morphological changes were observed by MtioTracker Red staining. Scale bar: 30 µm. All data were shown as mean ± SD (n = 3). #*p* < 0.05 versus control, *p < 0.05 versus IL-13 group as determined by one-way analysis of variance (ANOVA) and Dunnett’s *post hoc* test.

We used IL-13 to stimulate BEAS-2B epithelial cells and induce airway epithelial inflammation. The results showed that with the prolongation of IL-13 stimulation time, the expression of NOX4 gradually increased, suggesting that NOX4 may be involved in the regulation of epithelial inflammation (**Figure 2A**). Next, we knocked down NOX4 and then detected its effect on ROS. The results of flow cytometry showed that ROS levels increased significantly after IL-13 stimulation, while knocking down NOX4 inhibited ROS levels (**Figure 2B**). We further observed the effect of NOX4 knockdown on mitochondrial proteins. The results showed that mitochondrial division proteins Drp1 and p-Drp1 (Ser616) increased after IL-13 stimulation, and Drp1 showed a translocation from cytoplasm to mitochondria (**Figure 2C**). However, knocking down NOX4 inhibited Drp1 activation and mitochondrial translocation (**Figure 2C**). Subsequently, we further used the mitochondrial fission inhibitor Mdivi-1 to verify the effect of Drp1 on mitochondrial function. The results showed that Mdivi-1 significantly inhibited the expression and phosphorylation of Drp1 and prevented the mitochondrial translocation of Drp1 (**Figure 2D**). Mitotraker-red staining showed that the mitochondria of epithelial cells changed from normal filamentous to fragmented morphology after IL-13 stimulation, while the knockdown of NOX4 and Mdivi-1 treatments partially reversed the degree of mitochondrial fragmentation (**Figure 2E**).

### NOX4 and Drp1 Are Involved in Epithelial Cell Apoptosis and NLRP3 Activation

**Figure 3:**
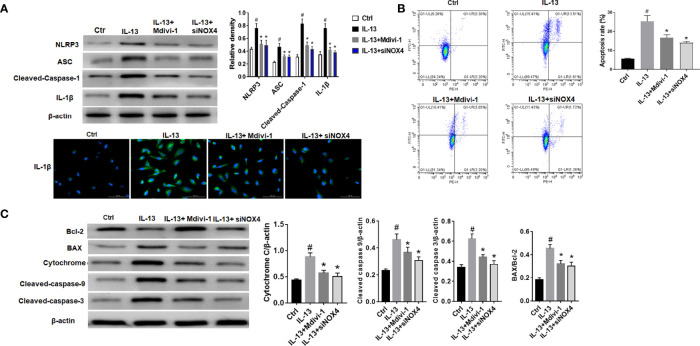
NOX4 and Drp1 are involved in epithelial cell apoptosis and NLRP3 activation. **(A)** Western blot of NLRP3, ASC, Cleaved-caspase-1 and IL-1β. Intervention was performed with siNOX4 and Mdivi-1, respectively, before IL-13 stimulation. IL-1β protein cytofluorogram. Green fluorescence represents IL-1β and blue fluorescence represents nucleus. Scale bar: 100 µm; **(B)** BEAS-2B cells were stimulated with IL-13. siNOX4 and Mdivi-1 were intervened in advance. Different apoptotic ratios were determined by flow cytometry analysis. **(C)** The expression of BAX, Bcl-1, cytochrome c, and Capsese-9/-3 was analyzed by Western blot. All data were shown as mean ± SD (n = 3). #*p* < 0.05 versus control, *p < 0.05 versus IL-13 group as determined by one-way analysis of variance (ANOVA) and Dunnett’s *post hoc* test.

The results showed that NLRP3 inflammasome was activated after IL-13 stimulation, including high expression of NLRP3, ASC and Cleaved-caspase-1 (**Figure 3A**). However, after treatment with Mdivi-1 or knockdown of NOX4, the expression of inflammasome-related proteins was significantly down-regulated, and the corresponding cell apoptosis was also significantly reduced (**Figure 3B**). Meanwhile, further examination of the expression of apoptosis-related proteins showed that after IL-13 stimulation, the level of anti-apoptotic protein Bcl-2 decreased, while the pro-apoptotic proteins BAX, Cytochrome C, Cleaved-caspase-9, and Cleaved-caspase-3 were significantly elevated (**Figure 3C**). Correspondingly, Mdivi-1 and siNOX4 both reversed the expression of these apoptotic proteins. The above results indicate that NOX4 may induce mitochondrial damage by regulating the expression of Drp1, thereby activating the NLRP3 inflammasome, causing epithelial cell inflammation and participating in epithelial cell damage.

### miRNA-182-5p Directly Targets NOX4

**Figure 4:**
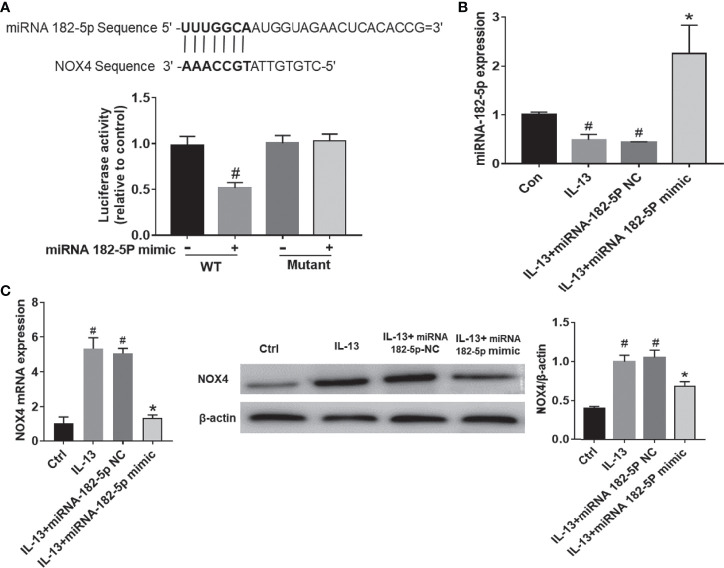
MiRNA-182-5p targets NOX4. **(A)** Prediction of binding sites. WT and mutant plasmids were co-transfected with miRNA-182-5p mimic or miRNA NC for dual-luciferase assay. #*p* < 0.05 versus wild-type control. **(B)** After transfection with miRNA-182-5p mimic, real-time PCR detected miRNA-182-5p expression. **(C)** After transfection with miRNA-182-5p mimic or NC, real-time PCR and Western blot were used to determine NOX4 mRNA and protein expression; All data were shown as mean ± SD (n = 3). #*p* < 0.05 versus control, *p < 0.05 versus IL-13 group as determined by one-way analysis of variance (ANOVA) and Dunnett’s *post hoc* test.

As predicted by miRbase website, there was a direct binding site of miRNA-182-5p on the 3’UTR of NOX4 (**Figure 4A**). To determine whether miRNA-182-5p directly regulates NOX4 gene expression, we further evaluated the binding of miRNA-182-5p to the 3’UTR of NOX4 gene by dual-luciferase reporter assay. The results showed that the miRNA-182-5p mimics significantly reduced the luciferase activity of the WT 3’UTR of NOX4, but not the mutant 3’UTR of NOX4. These results indicate that miRNA-182-5p binds to the 3’UTR of NOX4 mRNA and inhibits the expression of NOX4.

Next, we validated the expression of miRNA-182-5p after transfection of miRNA-182-5p mimic and miRNA-182-5p NC. (**Figure 4B**). The miRNA-182-5p expression was significantly decreased compared with the control group after IL-13 stimulation. Transfection with miRNA-182-5p mimic significantly increased the expression of miRNA-182-5p, indicating that miRNA-182-5p was overexpressed. Then, we measured the mRNA and protein levels of NOX4. The results showed that after IL-13 stimulation, the expression of miRNA-182-5p in BEAS-2B cells decreased, while the expression of NOX4 mRNA increased, overexpression of miRNA-182-5p suppressed NOX4 at both protein and mRNA levels (**Figure 4C**).

### miRNA-182-5p Is Involved in the Regulation of Mitochondrial Morphology

**Figure 5:**
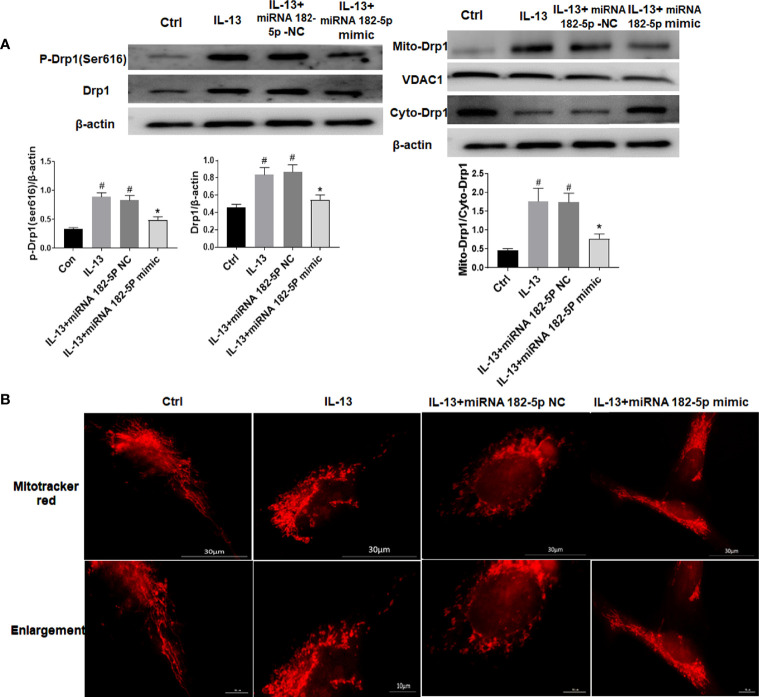
MiRNA-182-5p is involved in the regulation of mitochondrial morphology. **(A)** MiRNA-182-5p mimic or NC was used to intervene before IL-13 stimulation of BEAS-2B cells, and the expression of Drp1 and p-Drp1 (Ser616) was measured by Western blot. Drp1 translocation was observed by measuring the expression of Drp1 in the cytoplasm and mitochondria; **(B)** Mitochondrial morphological changes were observed by MtioTracker Red staining, Scale bar: 30 µm. All data were shown as mean ± SD (n = 3). #*p* < 0.05 versus control, *p < 0.05 versus IL-13 group as determined by one-way analysis of variance (ANOVA) and Dunnett’s *post hoc* test.

We tested the changes in mitochondrial fission protein after IL-13 stimulation. The results showed that the level of p-Drp1 (Ser616) increased significantly after IL-13 stimulation, and the total protein level of Drp1 also increased (**Figure 5A**). Moreover, Drp1 was transferred from the cytoplasm to the mitochondria. Next, we observed the morphology of mitochondria through the fluorescent dye Mitotracker-red (**Figure 5B**). We found that in normal cells, the mitochondria were elongated; but after IL-13 stimulation, the mitochondria appeared to be broken and fragmented. On the contrary, after transfection of miRNA-182-5p mimics, the expression levels of p-Drp1 (Ser616) and Drp1 protein decreased, and the translocation of Drp1 from the cytoplasm to the mitochondria was also inhibited. In addition, fluorescence imaging also showed that miRNA-182-5p reversed the degree of mitochondrial fragmentation.

### miRNA-182-5p Improves Mitochondrial Function

**Figure 6:**
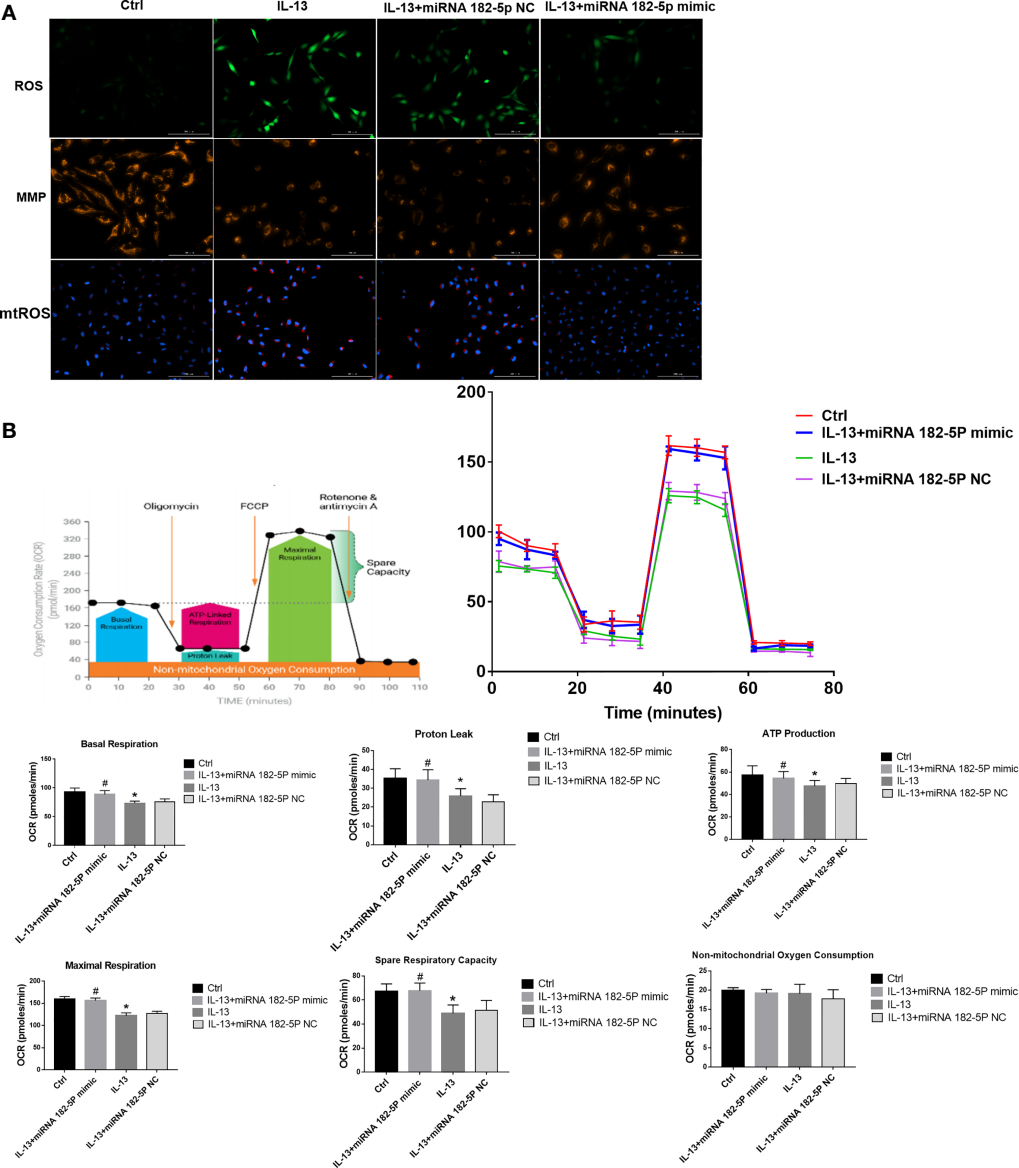
MiRNA-182-5p improves mitochondrial dysfunction. **(A)** MiRNA-182-5p mimic or NC was used to intervene before IL-13 stimulation of BEAS-2B cells. ROS Assay Kit, tetramethylrhodamine ethyl ester (TMRE) and MitoSOX ™ Red were used to stain BEAS-2B to observe the expression changes of ROS, MMP and mtROS, respectively. Green fluorescence represented ROS, orange fluorescence represented MMP and red fluorescence represented mtROS. Scale bar: 100 µm; **(B)** MiRNA-182-5p mimic or NC was used to intervene before IL-13 stimulation of BEAS-2B cells. The XFe hippocampal system was performed to determine the expression of proton leak, basal respiration, ATP production, spare respiratory respiration, and maximal perspiration in different groups. All data were shown as mean ± SD (n = 3). ^#^ *p* < 0.05 versus control, *p < 0.05 versus IL-13 group as determined by one-way analysis of variance (ANOVA) and Dunnett’s *post hoc* test.

Then, we further checked the mitochondrial function, including MMP, ROS, and mitochondrial respiration. The results showed that IL-13 treatment resulted in the up-regulation of intracellular ROS expression (**Figure 6A**) and triggered a decrease in the orange fluorescence of MMP, and an increase in the red fluorescence of mtROS. This indicates that IL-13 caused mitochondrial damage. After the intervention of miRNA-182-5p, the green fluorescence of ROS and the red fluorescence of mt-ROS were reduced, while the orange fluorescence of MMP was enhanced, suggesting that miRNA-182-5p inhibited mitochondrial damage. Further through the XFe Seahorse system, we examined the effect of miRNA-182-5p on mitochondrial respiration. After IL-13 stimulation, proton leakage, basal respiration, ATP production, reserve respiration capacity and maximum respiration were significantly reduced. After miRNA-182-5p intervention, the mitochondrial respiratory function was significantly restored (**Figure 6B**). These results indicate that miRNA-182-5p significantly alleviates IL-13-induced mitochondrial dysfunction.

### miRNA-182-5p Inhibits the Activation of NLRP3/IL-1β and Apoptosis

**Figure 7:**
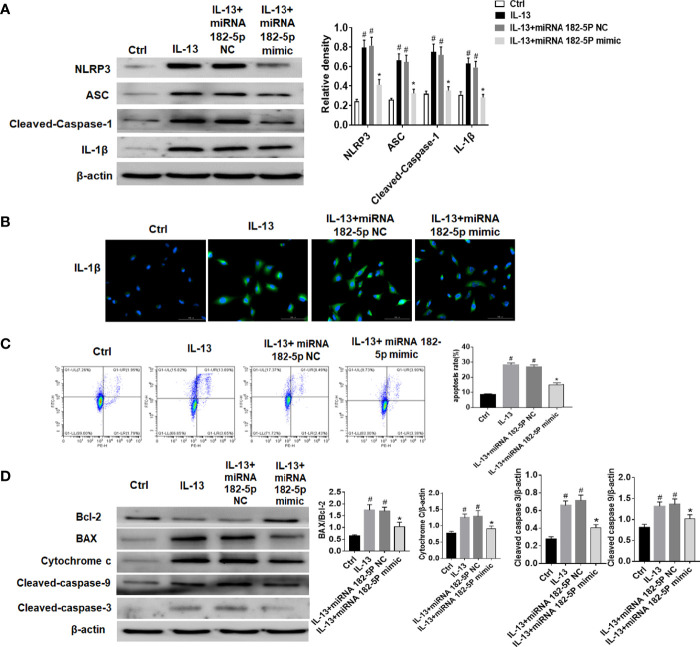
MiRNA-182-5p inhibits the activation of NLRP3/IL-1β and apoptosis. MiRNA-182-5p mimic or NC was used to intervene before IL-13 stimulation of BEAS-2B cells. **(A)** Western blot results of NLRP3, ASC, Cleaved-caspase-1 and IL-1β as well as IL-1β protein cytofluorogram were shown. **(B)** Green fluorescence represents IL-1β and blue fluorescence represents nucleus. Scale bar: 100 µm. **(C)** Different apoptotic ratios were determined by flow cytometry analysis. **(D)** The expression of BAX, Bcl-1, cytochrome c, Capsese-9 and 3 was analyzed by Western blot. All data were shown as mean ± SD (n = 3). #*p* < 0.05 versus control, *p < 0.05 versus IL-13 group as determined by one-way analysis of variance (ANOVA) and Dunnett’s *post hoc* test.

The NLRP3 inflammasome plays a key role in the pathogenesis of asthma (35, 36). Thus, we further verified the regulation of miRNA-182-5p on NLRP3/IL-1β. The results showed that the levels of inflammasome complex-related proteins including NLRP3, ASC and Cleaved-caspase-1 increased after IL-13 treatment. Correspondingly, IL-1β levels also increased significantly after IL-13 stimulation. However, after transfection of miRNA-182-5p mimics, NLRP3 inflammasome associated proteins were significantly reduced (**Figures 7A, B**). This indicates that miRNA-182-5p can inhibit NLRP3 inflammasome activation and IL-1β synthesis and reduce inflammation. Next, cell apoptosis was analyzed with flow cytometry. The results showed that IL-13 treatment significantly increased cell apoptosis (**Figure 7C**). However, after transfection of miRNA-182-5p mimics, the proportion of apoptosis was significantly lower than that in the IL-13 treatment group (*p <*0.05). The expression of apoptosis-related proteins was further detected by Western blot, which showed that after IL-13 stimulation, the level of anti-apoptotic protein Bcl-2 decreased, and the pro-apoptotic proteins including BAX, Cytochrome C, Cleaved-caspase-9, Cleaved-caspase-3 were elevated. On the contrary, miRNA-182-5p mimics reversed the changes of these proteins (*p <*0.05) (**Figure 7D**). The results indicate that miRNA-182-5p can suppress IL-13-induced mitochondrial apoptosis.

### miRNA-182-5p Inhibits Inflammation in Asthma Models

**Figure 8:**
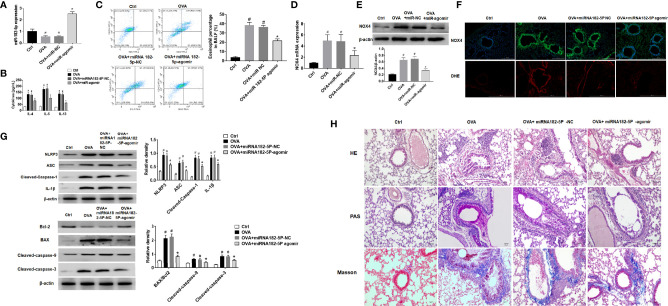
MiRNA-182-5p inhibits inflammation in asthma models. **(A)** Real-time PCR analyzed miRNA-182-5p level in lung tissues after injection of miRNA-182-5p agomir; **(B)** The expression of Th2-type cytokines IL-4, IL-5, and IL-13 in BALF was determined by ELISA; **(C)** The percentage of eosinophils in BALF of different groups was measured by flow cytometry. All data were shown as mean ± SD (n = 8). **(D)** Real-time PCR was used to determine NOX4 mRNA expression in different group; **(E)** Western blot was used to determine NOX4 protein expression in different group; **(F)** The expression of NOX4 and ROS in lung tissue was observed by immunofluorescence and DHE. Scale bar: 200 µm; **(G)** Western blot was used to measure NLRP3 inflammasome-associated proteins NLRP3, ASC, Cleaved-caspase-1 and IL-1β and apoptosis-associated proteins Bcl-2, BAX, Cleaved-caspase-9, and Cleaved-caspase-3 protein expression in lung tissues. **(H)** Lung tissue sections were stained with hematoxylin-eosin (HE) to observe inflammatory cell infiltration. Periodic acid-Schiff (PAS) was used to assess goblet cell hyperplasia. Sub-epithelial collagen deposition and fibrosis were assessed by Masson staining. Scale bar: 50 µm. All data were shown as mean ± SD (n = 3). #*p* < 0.05 versus control, *p < 0.05 versus OVA group as determined by one-way analysis of variance (ANOVA) and Dunnett’s *post hoc* test.

First, the expression of miRNA-182-5p in the lung tissue of animal model was verified by real-time PCR (**Figure 8A**). The expression of miRNA-182-5p decreased in the OVA group and the miRNA-182-5p NC group than control group, while miRNA-182-5p increased significantly after miRNA-182-5p agomir intervention, indicating that miRNA-182-5p agomir promoted the expression of miRNA-182-5p in lung tissues in the *in vivo* model. ELISA was used to confirm that miRNA-182-5p agomir significantly reduced the expression of Th2 inflammatory cytokines IL-4, IL-5, and IL-13 induced by OVA (**Figure 8B**). By flow cytometry, we further determined the effect of miRNA-182-5p agomir on the eosinophils in BALF. The percentage of eosinophils in BLAF in the OVA asthma model was significantly up-regulated compared with the control group, while the percentage of eosinophils in the miRNA-182-5p agomir group was significantly lower than that in the OVA group (*p <*0.05) (**Figure 8C**).

In OVA asthmatic mice, we verified the expression of NOX4 mRNA by real-time PCR. As in **Figure 8D**, there was high expression of NOX4 mRNA in the OVA group, while miRNA-182-5p agomir reduced the level of NOX4 mRNA. Then, the expression of NOX4 protein was further observed by Western blot and immunofluorescence, which obtained consistent results of NOX4 protein with those of NOX4 mRNA (**Figure 8E**). We further observed the expression of ROS in lung tissue by DHE staining. The results showed that the red fluorescence of ROS in the control group was weak, while in the OVA asthma group was obvious. However, after miRNA-182-5p agomir treatment, the ROS in lung tissue was largely decreased than that in the OVA group, indicating that miRNA-182-5p can reduce ROS in OVA asthma (**Figure 8F**). We also observed whether miRNA-182-5p inhibits the expression of NLRP3/IL1β proteins as well as apoptosis-related proteins in the OVA asthma model. As in **Figure 8G**, the expression of NLRP3 inflammasome-related proteins, including NLRP3, ASC, Cleaved-caspase-1 and IL-1β, were significantly increased after OVA treatment, while after miRNA-182-5p agomir intervention, their levels were significantly reduced. Next, we further tested the expression of apoptosis-related proteins (**Figure 8G**). The results showed that the expression of BAX and Cleaved-caspase-9/3 increased in the OVA asthma model, while the expression of Bcl-2 protein decreased, suggesting that there is excessive apoptosis in the OVA asthma model. However, after miRNA-182-5p agomir intervention, the levels of BAX, Cleaved-caspase-9 and Cleaved-caspase3 decreased significantly; meanwhile, the expression of Bcl-2 protein increased.

Finally, though HE, PAS, and Masson staining, we detected the pathological changes of lung tissue (**Figure 8H**). HE stainging showed that in the OVA-induced asthma model, the bronchial structure was destroyed, epithelial cells were detached, and inflammatory cell infiltration was obvious. However, treatment with miRNA-182-5p agomir evidently inhibited OVA-induced destruction of bronchial structures and infiltration of inflammatory cells around the airways and blood vessels. PAS staining showed that in the OVA group, there was obvious proliferation of goblet cells in the bronchus. Similarly, miRNA-182-5p agomir caused an obvious decrease in PAS-positive cells, indicating that OVA-induced goblet cell proliferation was reduced. In addition, Masson staining was used to detect airway and vascular collagen deposition. The results showed that a large amount of light blue stained collagen fiber deposition was observed under the bronchus and blood vessels in the OVA group, while miRNA-182-5p agomir evidently inhibited the deposition of collagen fibers under the bronchus and blood vessels. The above results indicate that miRNA-182-5p significantly improves airway inflammation in asthmatic mice.

## Discussion

In this study, we found the down-regulation of miRNA-182-5p in the OVA asthma model. *In vitro*, we used the TH2 cytokine IL-13 as a stimulant of epithelial inflammation and observed the role of miRNA-182-5p in the inflammatory response of epithelial cells. We found that IL-13 treatment resulted in a significant decrease in miRNA-182-5p in BEAS-2B cells. Then, we used miRNA-182-5p mimics to overexpress miRNA-182-5p in BEAS-2B cells. The use of miRNA mimics for miRNA overexpression was consistent with previous studies (37, 38). We verified the transfection efficiency by real-time PCR. We found that overexpression of miRNA-182-5p significantly reduced IL-13-induced apoptosis and expressions of NLRP3/IL-1β proteins. In addition, we further confirmed that miRNA-182-5p could directly target NOX4, reduce oxidative stress, inhibit the mitochondrial fission protein Drp1, and improve mitochondrial function. In animal models with asthma, we also found that miRNA-182-5p inhibited inflammatory cell infiltration, goblet cell proliferation and collagen deposition *in vivo*.

ROS is the key to airway epithelial cell dysfunction. Both NOX4-generated ROS and mtROS are the key to regulate oxidative stress. NOX4 is reported as a downstream target for miRNAs to regulate oxidative stress, indicating its key role in disease development (39, 40). In this experiment, the asthma model was simulated by OVA, and microarray showed that miRNA-182-5p was differentially expressed in asthmatic tissues. The possibility of miRNA-182-5p targeting NOX4 was predicted and verified by *in vitro* and *in vivo* experiments. Our results showed that miRNA-182-5p could inhibit the mRNA and protein expression of NOX4, thereby reducing the generation of ROS. Meanwhile, the decrease of NOX4 could affect the impairment of mitochondrial function, and the mechanism may be achieved by regulating mitochondrial dynamics.

Abnormal mitochondrial dynamics (fission/fusion) leads to mitochondrial dysfunction (20), which not only disrupts the integrity of the mitochondrial membrane leading to reduced membrane potential and affects mitochondrial energy metabolism, but also releases mtROS into the cytoplasm aggravating oxidative stress (41). Drp1 is a key regulatory protein during mitochondrial fission (42), and excessive Drp1 may promote the mitochondrial damage. In Alzheimer’s disease models, increased mtROS levels can lead to mitochondrial shortening and Drp1 activation (43, 44), which may lead to a vicious cycle. In *in vivo* experiments of this study, NOX4 was first observed to induce high expression of Drp1 and mitochondrial translocation. MiRNA-182-5p, although it did not directly target Drp1, was indirectly involved in mitochondrial injury by inhibiting NOX4.

Mitochondrial injury not only affects energy metabolism, but also promotes mitochondrial apoptosis. It has been pointed out that NOX4 drives apoptosis, which includes activation of the mitochondrial apoptotic pathway, and the damaged mitochondria can induce apoptosis by releasing cytochrome C (45). In this study, it was also observed that inhibition of NOX4 alleviated apoptosis, and miRNA-182-5p inhibited the apoptotic process by targeting NOX4/ROS to reduce the expression of mitochondrial apoptosis-related proteins. Mitochondrial damage is similarly involved in NLRP3 activation, while the correlation between NOX4 and NLRP3 is similarly confirmed. In dilated cardiomyopathy, Drp1 induced constitutive expression of NOX1 and NOX4 while promoted NLRP3 inflammasome activation through mitochondrial fission (46). In the study of diabetic nephropathy, knockdown of NLRP3 decreased the expression of thioredoxin-interacting protein and NOX4 and the production of superoxide in the diabetic kidney, and improved renal function (47). Thus, it can be seen that NOX4 affects mitochondrial dynamics, triggers mitochondrial injury, and activates the activation of NLRP3 by promoting the expression of ROS, while the activation of NLRP3 may further enhance the constitutive expression of NOX4 and aggravate oxidative stress-mitochondrial injury. NOX4 may be a key therapeutic target. MiRNA-182-5p directly targets NOX4 to affect the process of oxidative stress- mitochondria-apoptosis and NLRP3.

The *in vivo* model finally verified the effect of miRNA-182-5p on asthma. The miRNA-182-5p agomir was injected into the tail vein of animal for miRNA-182-5p agomir overexpression. MiRNA agomir is a miRNA agonist with special chemical modifications, is methylated and cholesterol modified on the basis of mimic and purified *in vivo*. It is more stable after methylation modification, can directly enter cells after cholesterol modification, does not cause animal death after *in vivo* purification, and can be directly injected into animals to mimic endogenous miRNA. After miRNA-182-5p agomir overexpression, we observed that miRNA-182-5p significantly improved the typical pathological changes of asthma, including reducing TH2 inflammatory factor expression, peri-airway inflammatory infiltration, goblet cell proliferation and collagen deposition. Another typical pathological feature of asthma is airway remodeling, and the proliferation of airway smooth muscle is the key to airway remodeling. This study did not elucidate the effect of miRNA-182-5p on airway smooth muscle cells, but it has been reported that miRNA-182-5p reduces pulmonary fibrosis through TGF (tumor growth factor)-β/Smad pathway (48). We suppose that miRNA-182-5p may delay the process of airway remodeling by affecting the expression of a variety of proteins. This needs to be further explored in the future.

In summary, miRNA-182-5p was significantly reduced in OVA asthma model. Further studies showed that NOX4 promoted ROS-induced oxidative stress and induced the accumulation of mitochondrial fission protein Drp1, which in turn promoted mitochondrial damage. Due to the activation of the mitochondrial apoptosis pathway and the activation of NLRP3, the inflammatory response of airway epithelial cells was finally promoted. Furthermore, we found that miRNA-182-5p directly regulated the expression of NOX4, and the overexpression of miRNA-182-5p significantly reduced the inflammatory response of airway asthma. Our results may provide new therapeutic targets for asthma.

## Data Availability Statement

The original contributions presented in the study are publicly available. This data can be found here: https://www.ncbi.nlm.nih.gov/geo/query/acc.cgi?acc=GSE197090.

## Ethics Statement

The animal study was reviewed and approved by the Ethics Committee of Yanbian University School of Medicine.

## Author Contributions

ZW: Study design, data collection, data interpretation, manuscript preparation. YS: Study design, data collection, manuscript preparation. JJ: Data collection. YP: Data collection. LL: Data collection. QB: Data collection. CX: Statistical analysis. HL: Data interpretation. LCL: Literature search. HP: Study design, literature search. GY: Study design, data interpretation, funds collection. All authors have read and approved the manuscript. All authors contributed to the article and approved the submitted version.

## Funding

This work was supported by National Natural Science Foundation of China (Nos.: 82060004, 81970018, 81860729 and 81660003).

## Conflict of Interest

The authors declare that the research was conducted in the absence of any commercial or financial relationships that could be construed as a potential conflict of interest.

## Publisher’s Note

All claims expressed in this article are solely those of the authors and do not necessarily represent those of their affiliated organizations, or those of the publisher, the editors and the reviewers. Any product that may be evaluated in this article, or claim that may be made by its manufacturer, is not guaranteed or endorsed by the publisher.

## Supplementary Material

Supplementary Figure 1 Schematic diagram of asthma model construction and miRNA-182-5p intervention.
