# Supplementary figures and images for: MicroRNA-182-5p Attenuates Asthmatic Airway Inflammation by Targeting NOX4
Source: Front Immunol. 2022 May 31;13:853848. doi: 10.3389/fimmu.2022.853848 (PMC9192947; doi:10.3389/fimmu.2022.853848)

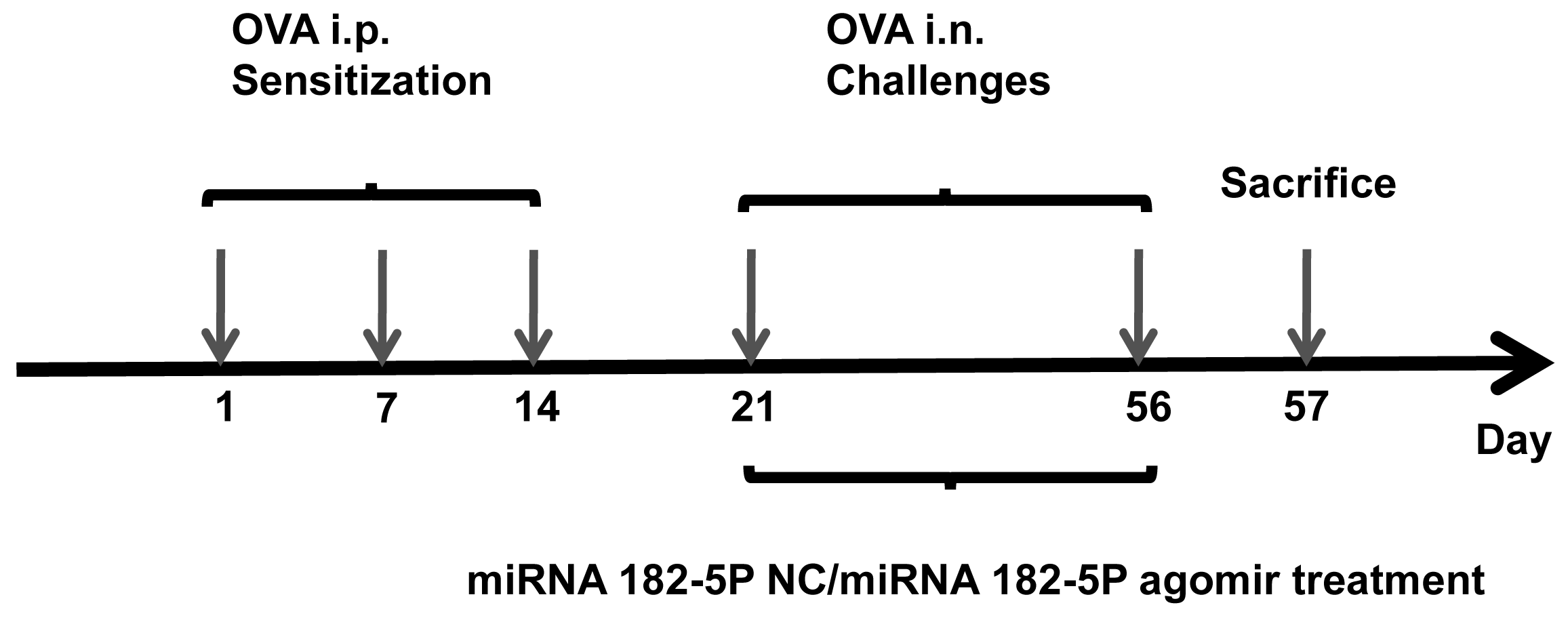

Supplement: Supplementary Figure 1 — Schematic diagram of asthma model construction and miRNA-182-5p intervention. [file Image_1.tif]
